# From crisis to opportunity: integrating insights from COVID-19 into the autism research

**DOI:** 10.3389/fpsyt.2024.1341737

**Published:** 2024-02-09

**Authors:** Chiara Failla, Paola Chilà, Noemi Vetrano, Germana Doria, Ileana Scarcella, Roberta Minutoli, Stefania Gismondo, Giovanni Pioggia, Flavia Marino

**Affiliations:** ^1^ Institute for Biomedical Research and Innovation (IRIB), National Research Council of Italy (CNR), Messina, Italy; ^2^ Classical Linguistic Studies and Education Department, Kore University of Enna, Enna, Italy; ^3^ Faculty of Psychology, International Telematic University Uninettuno, Roma, Italy; ^4^ Department of Cognitive, Psychological Science and Cultural Studies, University of Messina, Messina, Italy

**Keywords:** autism, telehealth, COVID-19, parent, well - being

## Introduction

The pandemic COVID-19 pandemic has presented substantial obstacles to families around the world. Several countries have implemented preventative measures, leading notable disturbance in daily routine. Lockdowns, which have limited physical attendance at school, required parents to adapt to remote working and strongly discouraged social interactions, have radically altered the family landscape. Research indicates that the pandemic has the potential to elicit greater psychological distress in children with neurodevelopmental disorder, like autism spectrum disorders (ASD) (1). The concept of vulnerability, acknowledged by the World Health Organization, plays a crucial role in the context of people with ASD (2). The distinctive difficulties in communication, socialization and executive functions associated with ASD have made this segment of the population more fragile during the pandemic (3). Consequently, these children thrive in highly structured environments and unexpected changed can induce stress, anxiety, or confusion, as noted by Baron-Cohen (4).

Preventive measures implemented during the last global health crisis, have exerted an influence on the well-being of ASD population. This impact is further accentuated by the high incidence of concurrent mental health issues (5), disruptions to daily routines and reduced access to essential support systems. Some children with ASD have experienced a decline in stress levels as a result of fewer situation requiring social interactions and environmental demands potentially resulting in enhancements in their emotional and behavioral well-being (6, 7). A global initiative to transition health services to a “remote by default” model has been in place since March 2020 in response to the pandemic (8). Telemedicine has become a central focus, constantly to address new clinical demands through digital advancements. Telehealth promotes the relationship between patients and doctors while alleviating pressure on healthcare systems (9). Technology-mediated care, including real-time video calls, health monitoring via medical devices, email, audio and instant messaging, virtually connects doctors with patients or caregivers, eliminating the need for physical proximity. This strategy has also been employed to provide assistance to ASD families.

## Effect of healthcare service

As highlighted in their study conducted by Zerbo, individuals with ASD require higher levels of service utilization and incur greater costs compared to those with other disabilities (10). However, compelling evidence highlights substantial unmet healthcare needs existing even prior to the pandemic. Preceding the pandemic, people with ASD already experienced disruptions in the services they regularly used, attributed to factors such as staff turnover, shortages, mandated closures of community resources, and significantly altered or reduced access to essential specialized services (11). Telemedicine is effective and preferred by some patients in behavioral health services (12, 13). The benefits of telemedicine extend beyond mere convenience, particularly given the severe limitations on the availability and capacity of in-person care during the pandemic. Telemedicine emerges as a key element, reducing costs and improving geographic accessibility for patients, particularly for children with developmental disabilities, by involving healthcare workers and siblings (14).. Telehealth has become a vital resource, bridging the gap left by the constrained capabilities of providers and health systems. However, research in the scientific landscape on the sustainability of telemedicine for individuals with ASD presents a mixed picture (15). While young people with ASD and their parents generally perceive telemedicine positively, technical issues pose a concern in the delivery of these services (16). Despite these challenges, telemedicine proves to be a valuable solution in the field of autism.

## Telehealth and autism

The utilization of telemedicine in the context of autism has proven to be particularly impactful, especially during the years of the pandemic. In the study by Narzisi 2020 (17), the importance of clear communication about COVID-19 to children, emphasizing structured routines and suggesting the use of games, such as Lego therapy, to improve social cognition in children with ASD and to propose rules for sharing video games and the Internet, reducing social isolation and encouraging shared activities based on common interests between parents and children. This method of intervention has demonstrated remarkable effectiveness in enhancing various skills in children with autism. The remote delivery of services, including individual therapy, family counseling, and support for skill development, has been instrumental in overcoming the challenges posed by in-person restrictions. The adaptability of telehealth has not only facilitated consistent access to necessary interventions but has also showcased its potential to effectively address the unique needs of individuals with autism, providing a valuable avenue for improvement in diverse aspects of their development (18). A review study conducted by Courtney L. et al. in 2023 (19) explored the use of telemedicine for interventions with individuals with ASD. These results indicate that telemedicine has led to positive outcomes, involving active engagement of parents in clinical intervention practices with their ASD children. Additionally, direct involvement of ASD children in on line therapy sessions has been associated with positive outcomes, emphasizing the potential benefits of telemedicine for both parents and children. Several studies have consistently demonstrated the validity of telemedicine in the context of autism, encompassing both direct interventions with children (20–22) and programs involving active parental engagement aimed at teaching skills to enhance their children’s adaptive capabilities (23–25). The evidence underscores the effectiveness of telemedicine as a valuable and versatile tool for delivering targeted interventions, promoting skill development, and fostering adaptive behaviors in children with autism. This approach not only extends the reach of intervention strategies but also empowers parents to actively participate in their children’s developmental journeys, highlighting the potential of telemedicine as a comprehensive and accessible avenue for supporting families of autistic children. Furthermore, looking to the future, as highlighted by Narzisi A., 2020 (26) telemedicine could be useful for quicker diagnoses of autism, overcoming long waiting lists and integrating with conventional methods to guarantee early diagnoses that have a positive impact on the development path of children with ASD, as the effectiveness of the use of this modality has also been demonstrated in the diagnostic field.

## Discussion

During periods of emergency due to sudden disasters, individuals with disabilities experience significant challenges (27). Given the heightened vulnerability of individuals with ASD in such situations, it is essential to proactively work on preparing for these individuals who are more susceptible to managing emergency periods. Clinical services supporting individuals with ASD experienced a significant disruption due to the COVID-19 pandemic peak (28). To address this shortage of service, health systems promptly mobilized to compensate for the clinical support services that could be provided to the population. Telemedicine has provided the opportunity to fill the gap caused by suspension of in-person therapies that were impossible to conduct during the most acute phases of the pandemic. Ensuring continuity in support services can help individuals navigate the process of adapting to changes in their environment. Telemedicine is a valuable resource that needs to be expanded, accompanied by adequate dissemination of information about its usage (29). Interventions provided during the pandemic era have witnessed a high level of collaboration among families, caregivers and clinicians to facilitate better adaptation for individuals with ASD. A crucial aspect during this period has been addressing responses to anxiety and uncertainty stemming from a situation that disrupted every pre-established pattern of life (30). Likewise, individuals with ASD might endure signs of immediate stress or post-traumatic stress disorder (PTSD) beyond the active pandemic duration (31). Over the past decade, there has been the creation of training initiatives aimed at supporting community first responders in identifying and defusing situations involving distressed individuals with autism (32–34). For the future, it would be beneficial to develop plans and strategies in anxiety-free situations, to identify appropriate coping strategies for dealing with unexpected events. Learning such strategies in anxiety-free situation has been shown to facilitate the acquisition of these skills. An approach focused on finding valid alternative strategies could be a winning way to promote a sense of independence in individuals (35).

In conclusion, it is crucial to reflect on the main challenge faced by individuals with ASD during the pandemic, which is the adaptation to a new way of approaching daily life, breaking established patterns (36). Clinical operators have played a fundamental role in managing this global crisis, effectively providing support to a more vulnerable segment of the population and equipping them with the necessary tools to adapt better to everyday life. Healthcare professionals have a unique opportunity to cultivate fundamental skills in individuals with autism through everyday interactions, focusing on problem-solving, identifying alternative choices, and predicting outcomes. This sustained commitment during non-crisis moments is essential for building resilience and fostering confidence in managing uncertainty (37). Lessons learned from the pandemic underscore the importance of proactive intervention through telemedicine, providing vital support for the ongoing development of these essential skills in individuals with autism. Telehealth, when integrated into ongoing care strategies, not only addresses immediate challenges but also establishes the foundation for long-term resilience and adaptability in individuals with autism.

## Author contributions

CF: Conceptualization, Writing – original draft, Writing – review & editing. PC: Writing – original draft, Writing – review & editing, Conceptualization. NV: Writing – original draft. GD: Writing – original draft. IS: Writing – original draft. SG: Project administration. RM: Supervision, Writing – review & editing. GP: Project administration, Supervision, Writing – review & editing. FM: Project administration, Supervision, Writing – review & editing.
